# Tunable Magnetocaloric Effect in Ni-Mn-Ga Microwires

**DOI:** 10.1038/s41598-018-35028-9

**Published:** 2018-11-08

**Authors:** Mingfang Qian, Xuexi Zhang, Longsha Wei, Peter Martin, Jianfei Sun, Lin Geng, Thomas Bligh Scott, Hua-Xin Peng

**Affiliations:** 1 School of Materials Science and Engineering, Harbin Institute of Technology, Harbin, 150001 P.R. China; 2 Advanced Composites Centre for Innovation and Science (ACCIS), University of Bristol, Bristol, BS8 1TR UK; 3 Interface Analysis Centre (IAC), University of Bristol, Bristol, BS8 1TL UK; 4 Institute for Composites Science Innovation (InCSI), School of Materials Science and Engineering, Zhejiang University, Hangzhou, 310027 P.R. China

## Abstract

Magnetic refrigeration is of great interest due to its high energy efficiency, environmental friendliness and low cost. However, undesired hysteresis losses, concentrated working temperature interval (*WTI*) and poor mechanical stability are vital drawbacks that hinder its practical application. Off-stoichiometric Ni-Mn-Ga Heusler alloys are capable of giant magnetocaloric effect (MCE) and tunable transformation temperatures. Here, by creating Ni-Mn-Ga microwires with diameter of 35–80 μm using a melt-extraction technique, negligible hysteresis and relatively good mechanical stability are found due to the high specific surface area (SSA) that reduces incompatibility between neighboring grains. The high SSA also favors the element evaporation at high temperatures so that the transformation temperatures can be feasibly adjusted. Tunable magnetocaloric effect owing to different magneto-structural coupling states is realized by (i) composition design and subsequent tuning, which adjusts the temperature difference between the martensite transformation (MT) and the magnetic transition, and (ii) creation of gradient composition distribution state, which manipulates the MT range. Magnetic entropy change *ΔS*_*m*_ ~−18.5 J kg^−1^ K^−1^ with relatively concentrated *WTI* and *WTI* up to ~60 K with net refrigeration capacity ~240 J kg^−1^ at 50 kOe are demonstrated in the present Ni-Mn-Ga microwires. This criterion is also applicable for other small-sized materials.

## Introduction

Room temperature magnetic refrigeration has attracted significant interests recently due to its high energy efficiency, good environmental friendliness and low cost, which may potentially act as an alternative to the conventional vapor-compression technology^1^. The magnetocaloric effect (MCE), an adiabatic change in temperature or isothermal change in entropy as a response of a magnetic field change, has been extensively studied in various type of prospective candidate materials, among which the Heusler-type Ni-Mn-based compounds are of great interests due to their tunable martensite transformation (MT) temperature and ability to achieve giant magnetic entropy change (*ΔS*_*m*_)^2–11^. However, several drawbacks such as undesired hysteresis loss, concentrated working temperature interval (*WTI*) and poor mechanical stability are accompanied that hinder their practical applications.

Discontinuous volume change between the parent and transformed phases is a signature of the first-order transformation (FOT). The volume change may hinder the structural transition due to the constraints between neighboring grains, thus, results in high transition hysteresis^12^. Ni-Mn-Ga alloys, during cooling, undergo MT from a cubic L2_1_ structure (austenite) to a complex tetragonal or orthorhombic structure (martensite), accompanied by a volume change of about 0.7–0.9% (volume per atom)^13^. In this context, high ratio of grain free surfaces would reasonably release most of the volume change so that the constraints caused by neighboring grains can be effectively reduced, thus leading to reduced hysteresis during phase transition. Furthermore, the hysteresis loss during MT is also related to the interfacial motion, e.g. martensite/austenite phase boundary, twin boundary and magnetic domain wall, which would be improved with the reduction of the neighboring grain constraints^14^. On the other hand, Ni-Mn-Ga alloys exhibit a high intergranular fracture tendency because of the high elastic anisotropy, long-range ordered L2_1_ crystal structure and mixed metallic-covalent bond, giving rise to a low mechanical stability^15^. The increased free surface also favors the strain energy release between grains, which is fundamental for the enhancement of mechanical stability. Porosities have been introduced into bulk alloys to reduce the hysteresis loss and brittle fracture tendency by reducing the grain boundary density, however, it would somehow “dilute” the refrigeration efficiency^16^. Our previous work found that magnetic hysteresis can be reduced due to the removal of grain boundaries that restrain volume change and interfacial motion in stress relief annealed single crystalline Ni-Mn-Ga micro-particles^8^. The hysteresis during FOT is also related to the internal stresses or defects, which can be diminished by annealing heat treatment^13^.

Ni-Mn-Ga alloys exhibit tunable MT temperatures where the combined structural and magnetic contributions make them promising magnetic refrigeration materials^17^. Generally, according to the Maxwell equation, a higher value of ∂M/∂T implies a higher MCE, thus, a first-order magnetic transition (FOMT) giving rise to an abrupt change of the magnetization (*ΔM*) in the vicinity of the transition point is responsible for a giant MCE^10^. Therefore, due to the tunable transition temperatures of Ni-Mn-Ga alloys, giant MCE can be achieved when the MT (a first-order transformation, FOT) is tuned to be coupled with the magnetic transition (a second-order transition, SOT)^2,3,11^. However, the first-order character of the transition sharpens the response so that the high *ΔS*_*m*_ usually concentrated in a very narrow temperature range, 1–3 K^2,11^, which is obviously not promising for practical applications because an applicable magnetic refrigerator requires a wide temperature span of the MCE. Particularly, for an Ericsson-type refrigerator, a constant *ΔS*_*m*_ through a wide thermodynamic cycle range is required^18^. Enhancement of the *WTI* has been realized by mixing several materials with different transition temperatures into a composite, successive *ΔS*_*m*_ peaks, and thus a broadened *WTI* could be anticipated^19^. However, the reduction of the magnitude of *ΔS*_*m*_ with the increasing number of constituent materials would become an unavoidable hindrance for the practicability of this approach^19,20^. Efforts have also been made in monolithic materials by adjusting successive second-order transitions or introducing intermediate phase transitions, while the *WTI* obtained was either with limited expansion or at extremely low temperature region^4,21^. Furthermore, the production of magnetocaloric components used as regenerators in a magnetic refrigeration device is a significant challenge. Considering the high heat exchange efficiency, the high specific surface area (SSA) of small-sized materials may increase the contacting area between the materials and the heat-transfer agent, which enhances the magnetic refrigeration efficiency.

Here, we present a solution with respect to the abovementioned several drawbacks by effectively synthesizing the microscale-diameter microwires using a melt-extraction technique^13,22^. Negligible hysteresis and reasonably good mechanical stability are attained. The high SSA of the microwires favors the element evaporation at high temperatures so that the MT temperature can be feasibly adjusted, resulting in the tunable MCE in the microwires. Large magnetic entropy change *ΔS*_*m*_ ~ −18.5 J kg^−1^ K^−1^ was achieved when the FOT and SOT were tuned to be overlapped. Of note is that significant improvements of the *WTI* ~60 K as well as refrigerate capacity (*RC*) ~240 J kg^−1^ were achieved by the combinational tuning effects of: (1) composition tuning, which adjusts the temperature difference between FOT and SOT (*ΔT*_*F-SOT*_), and (2) gradient composition distribution state tuning, which enlarges the transition range of the FOT (i.e. *M*_*s*_ − *M*_*f*_ or *A*_*f*_*−A*_*s*_, where *M*_*s*_, *M*_*f*_, *A*_*s*_ and *A*_*f*_ are the start and finish temperatures of the forward and reverse MT, respectively) and thus creates a widened *ΔS*_*m*_ peak through partial overlap of the FOT and SOT. The evolution of the MCE with respect to *ΔT*_*F-SOT*_ and the transition range is schematically illustrated (Supplementary Information Fig. S1). The small-sized microwires may be directly used in microscale devices, or they can act as building blocks for assembling complex shaped magnetic refrigerant devices^23^.

## Results and Discussion

**Figure 1:**
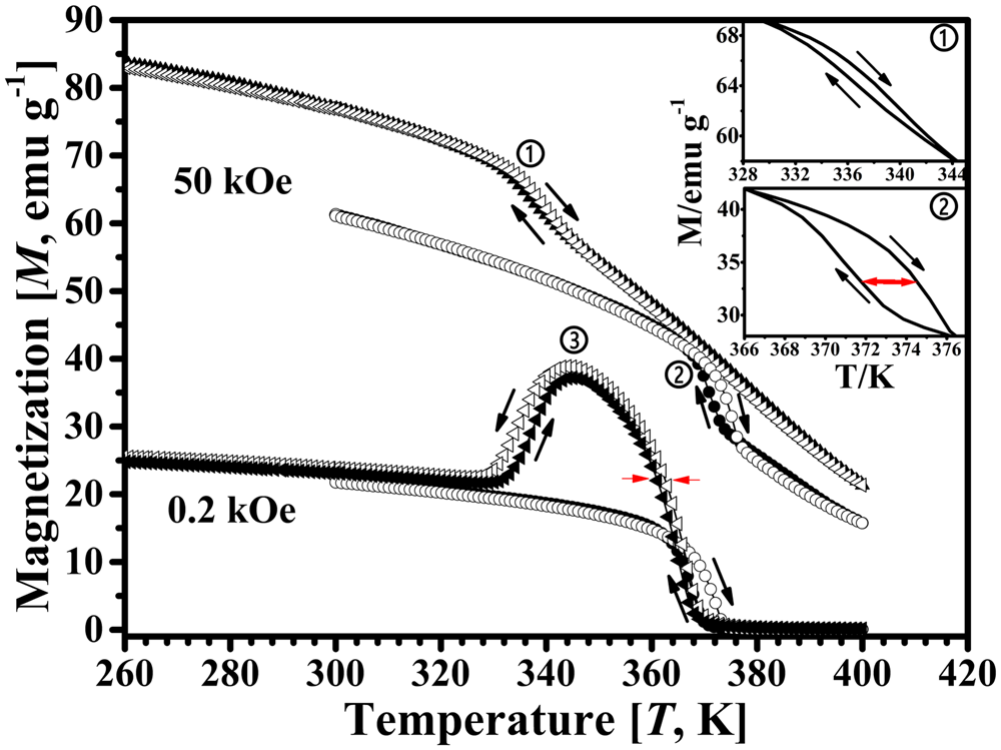
Isofield magnetization *M*(*T*) curves recorded at H = 0.2 and 50 kOe upon cooling (filled symbols) and heating (open symbols) for magneto-structural coupled W1 (circles) and partly coupled W2 (triangles). Insets ① and ② show the magnification of the corresponding areas. The red double-headed arrow in ② indicates the thermal hysteresis which is the largest width between the heating and cooling curves. ③ indicates the intersection point between the first- and second-order transformations of W2.

Ni-Mn-Ga microwires, with length of 30–200 mm and diameter of 35–80 μm, were fabricated on a large scale^22^ with D-shaped cross-section accompanied by columnar grains growing along the radial direction (Supplementary Information Fig. S2). The microstructure details of the melt-extracted Ni-Mn-Ga microwires can be found elsewhere^13^. Assuming that a microwire has a diameter of 57.5 μm and length 115 mm, the SSA of the microwires is ~7.8 times higher than that of the cubic bulk alloy with the same mass. Rely on the high SSA of the microwires, low transition hysteresis and relatively good mechanical stability are expected. Figure 1 shows the isofield magnetization *M*(*T*) curves recorded at *H* = 0.2 and 50 kOe upon cooling and heating for the magneto-structural coupled W1 and partly coupled W2. The thermal hysteresis, i.e. the temperature difference between the heating and cooling curves during MT (denoted as red double headed arrows in Fig. 1), is significantly suppressed to ~3.7 K (~2.8 K) in W1 and ~3.1 K (~1.1 K) in W2 at magnetic field of 0.2 kOe (50 kOe) (inset in Fig. 1), respectively, which is much smaller than that of bulk Ni-Mn-Ga alloys, ~10 K^12^.

**Figure 2:**
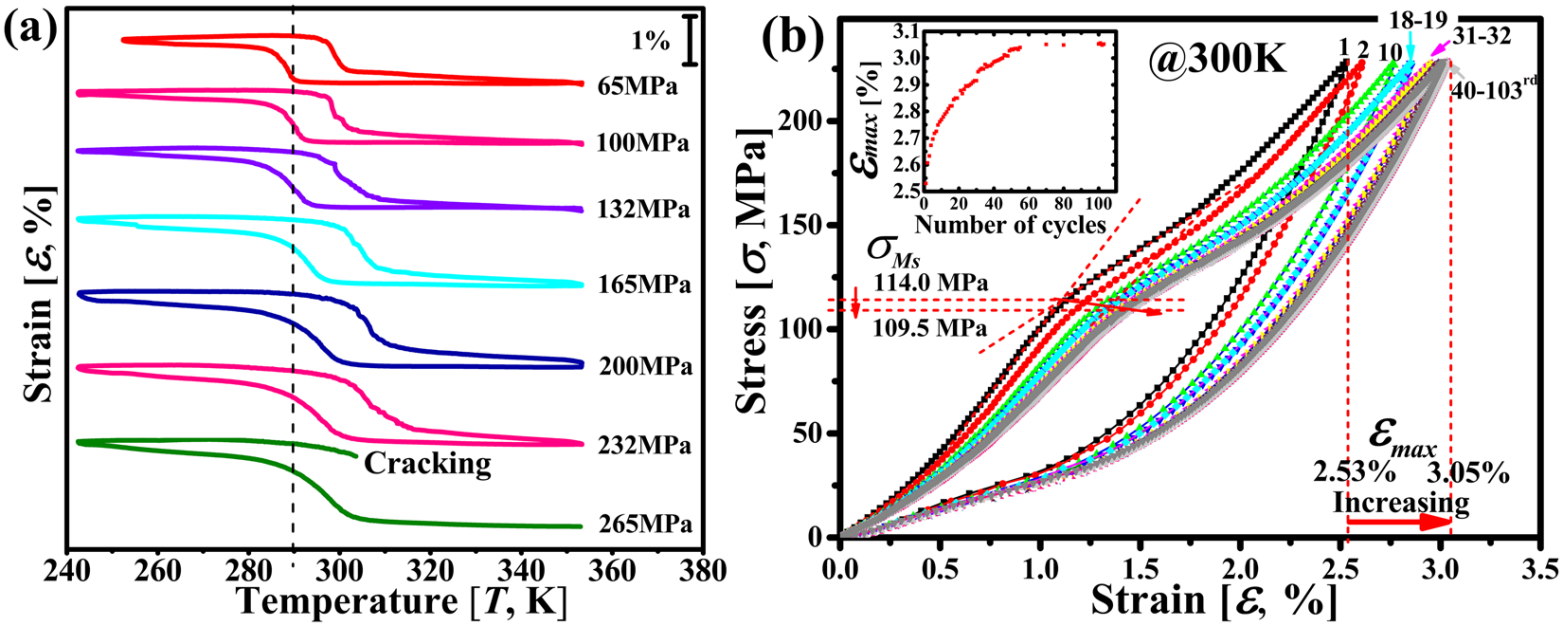
Mechanical properties of the microwires. (**a**) Stress-assisted thermal cycling curves and (**b**) superelastic loop curves of Ni-Mn-Ga microwires (W3), the inset shows the maximum superelastic strain *ε*_*max*_ versus the number of superelastic cycles. Different stresses from 65 to 265 MPa were applied during thermal cycling in (**a**). The MT temperatures increase with increasing external stress (the black dash line in (**a**) shows the difference between *M*_*s*_ at 65 MPa and at other stresses). The critical stress for stress-induced MT transformation *σ*_*Ms*_ (defined as the intersections of the lines extrapolated from the elastic curve and the forward transition region) reduces and the maximum strain *ε*_*max*_ increases during cycling in (**b**). This is mainly due to the “training” effect induced by the superelastic cycling, during which the stress induced martensite is inclined to form along preferred orientations with fewer variants, thus, reducing the required stress and enhancing the strain. This phenomenon becomes less obvious with increasing number of cycles.

Large deformation in Ni-Mn-Ga alloys can be observed during a stress-induced MT (known as superelasticity) or due to the movement of twin boundaries^13,14^. However, the Ni-Mn-Ga alloys show intrinsic brittleness which is easy to cause intergranular fracture thus limits their applications. Considering, in comparison to serving under a cyclic external magnetic (as in a magnetic refrigeration device), the brittle fracture tendency of the Ni-Mn-Ga alloys would be much more severe when a stress-assisted thermal cycling (across the MT region) or a stress-induced MT is introduced. Thus, to evaluate the mechanical stability, thermal cycles were performed under various tensile stresses in the present work, as shown in Fig. 2a. The results show that low stress (i.e. <100 MPa) was unlikely to cause fracture, where more than 100 times of cycles were performed (not shown). However, the microwire may fracture during the thermal cycling when the stress exceeds certain value, in this case, 265 MPa. Furthermore, the superelastic loop test was performed at 300 K with a maximum stress value of 232 MPa for 103 times, as shown in Fig. 2b. It shows that the microwire maintained unbroken after >100 times of loading-unloading cycles. We believe that this relatively good mechanical stability in comparison with bulk alloys is attributed to the high SSA that reduced constraints and internal stress between neighboring grains during MT in the one-dimensional microwires.

**Figure 3:**
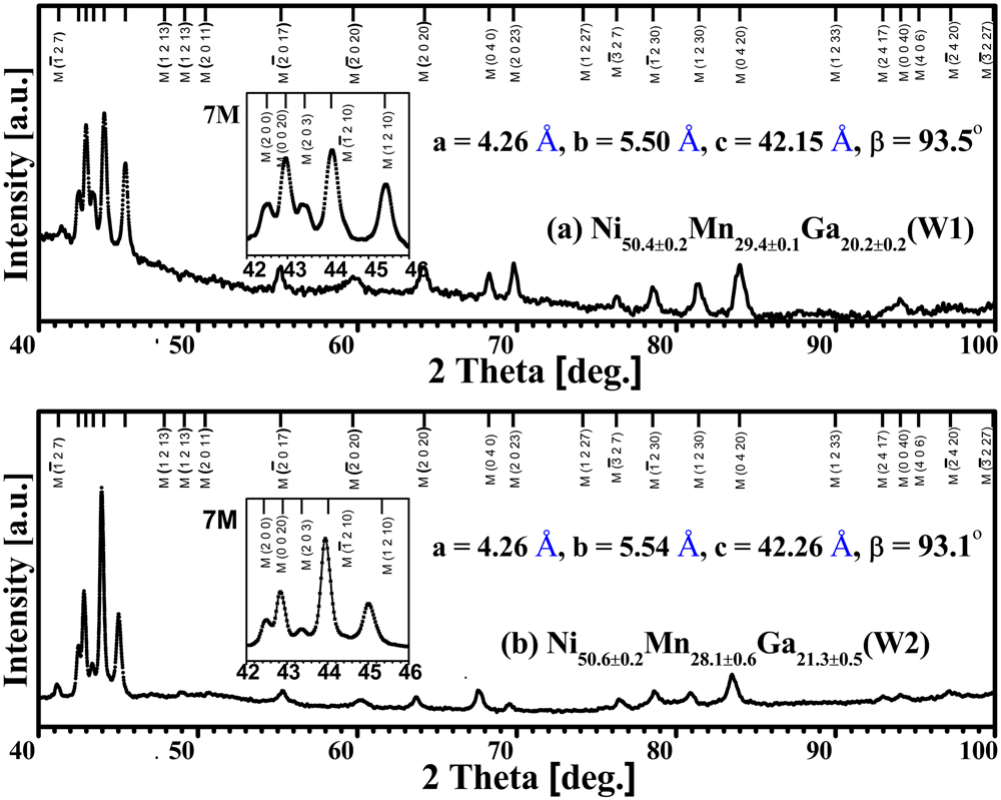
Room temperature X-ray diffraction (XRD) patterns of (**a**) W1 and (**b**) W2. The insets show diffraction peaks between 42° and 46° of the microwires.

Figure 3 shows the room temperature XRD patterns of the present microwires. From the *M*(*T*) curves in Fig. 1, it is noted that the magnetic and structural transitions occur simultaneously in W1. Together with the structure characterization, as shown in Fig. 3a, one can find that the paramagnetic to ferromagnetic transition is coupled with a single austenite to a 7M martensite structural transition for W1. The electron diffraction pattern confirms the 7M structure of W1 (Supplementary Information Fig. S3). By the whole pattern fitting analysis, the 7M martensite is determined to be a monoclinic incommensurate superstructure^24^ with the crystal lattice constants *a* = 4.26 Å, *b* = 5.50 Å, *c* = 42.15 Å, *β* = 93.5°.

The reduced hysteresis and relatively good mechanical stability make Ni-Mn-Ga microwires a potential practical refrigerant material where a large *ΔS*_*m*_ is required. This magneto-structural coupling, i.e. the overlap of the magnetic and structural transitions, gives rise to a large change in the distance between the magnetic atoms (i.e. Mn) in the crystal lattice during the magnetic transition which, in return, results in a significant *ΔM* in the vicinity of the transition^3^. An abrupt and relatively large *ΔM* of ~16 emu g^−1^ under 0.2 kOe is observed at the transition area around ~368 K with a *dM*_*p*_*/dH* (Sensitivity of the MT temperature to the applied field) of ~0.06 K kOe^−1^ (Supplementary Information Table S1 and Fig. S4a,b) in W1, implying the existence of the magnetic field induced transformation from the paramagnetic austenite to the ferromagnetic martensite. On the other hand, the magnetization of the ferromagnetic phase was found to increase after annealing due to the increased atomic ordering degree, which contributes to the large *ΔM* and influences the *ΔS*_*m*_ of Ni-Mn-Ga alloys^13^.

The *ΔS*_*m*_ was calculated from the magnetic isotherms (Supplementary Information Fig. S5) using the Maxwell relation (Equation (1)). The validity and reliability of the Maxwell relation in evaluating the MCE in Ni-Mn-Ga alloys has been confirmed due to its weak magneto-elastic coupling during FOT^10^.

1 $${\rm{\Delta }}{S}_{m}{(T,H)}_{{\rm{\Delta }}H}={\int }_{0}^{{H}_{{\rm{\max }}}}{(\frac{\partial M}{\partial T})}_{H}dH$$

**Figure 4:**
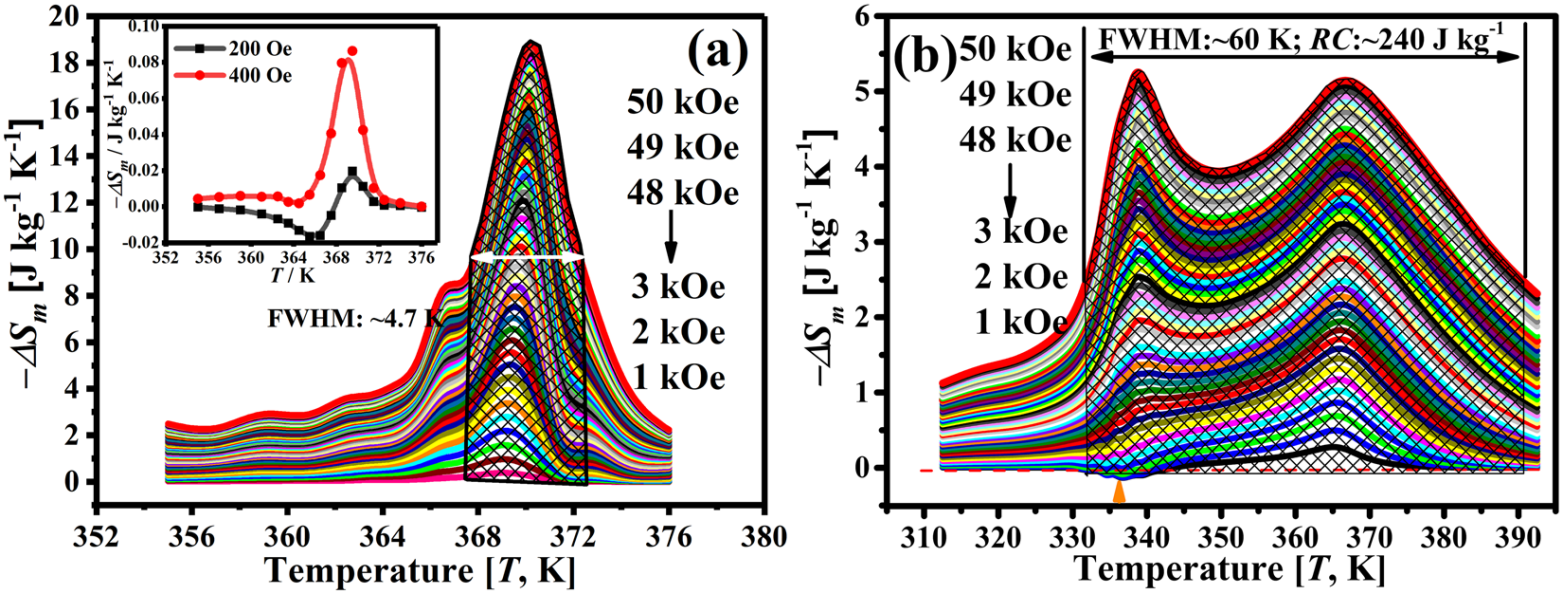
Temperature dependence of magnetic entropy change *ΔS*_*m*_(*T*) of (**a**) W1 and (**b**) W2 under different magnetic fields derived from the isothermal magnetization curves. The inset in (**a**) demonstrates the *ΔS*_*m*_(*T*) at 200 and 400 Oe of W1. The white double sided arrow in (**a**) indicates the full width at half maximum of W1. The orange arrow in (**b**) marks the tiny positive entropy peak.

Figure 4a plots the temperature dependence of the calculated *ΔS*_*m*_(*T*) under different magnetic fields. The *ΔS*_*m*_ reaches the maximum level around the magneto-structural transition temperature. The *ΔS*_*m*_ peak temperature exhibits a field dependence behavior, i.e. it shifts to a higher temperature with increasing magnetic field (from ~368.5 to ~370.5 K). Besides, as shown in the inset in Fig. 4a, a small positive peak of ~0.02 J kg^−1^ K^−1^ at ~366 K under an extreme low magnetic field 200 Oe was detected. It is related to the magnetic domains which are stabilized with preferred orientation and thus are hard to be magnetized under a low magnetic field. It has been reported that both critical field and positive peak values decrease with increasing *e/a*, that is, as *T*_*c*_ − *M*_*p*_ goes to zero^25^. Therefore, both values are rather small in W1 due to the overlap of the FOT and SOT (*e*/*a* = ~7.7). However, the small positive peak evolved into small bumps on the left side of the *ΔS*_*m*_ peak with increasing magnetic fields, as shown in Fig. 4a, indicating the subtle difference between *T*_*c*_ and *M*_*p*_. The maximum *ΔS*_*m*_ of ~−11.3 and ~−18.5 J kg^−1^ K^−1^ were obtained at magnetic fields of 20 and 50 kOe, respectively, which are comparable to that of polycrystalline Ni-Mn-Ga ribbons, films and bulk alloys^2,4,26^. However, concentrated *WTI* value (FWHM, half maximum at full width) ~4.7 K was attained at 50 kOe.

Nevertheless, to evaluate a refrigeration material, aside from the magnitude of the *ΔS*_*m*_, a wide *WTI* with high *RC* value is attractive and practical as well. As mentioned earlier, a solution of increasing the *ΔT*_*F-SOT*_ and widening the FOT temperature range by virtue of FOT and SOT partial overlap are feasible to broaden the *WTI*. A *ΔT*_*F-SOT*_, i.e. the temperature difference between the *M*(*T*) curve first derivative extreme values of FOT (337 K) and SOT (365 K), ~28 K in W2 is attained (Supplementary Information Fig. S4c). On the other hand, due to the high SSA of the microwires and evaporation of Mn element at high temperatures, a gradient Mn distribution state, i.e. depletion of Mn near the surface layer on the cross-section, was obtained by performing a vacuum annealing heat treatment (Supplementary Information Fig. S6). The variation of Mn content from the surface to the inner part leads to a widened MT temperature range |*A*_*f*_ − *A*_*s*_| in W2, ~15.2 K (Supplementary Information Table S2). As the Curie temperature of the austenite (*T*_*c*_^*A*^) keeps almost intact^12^, therefore, these two factors resulted in a partly overlapped state between FOT and SOT, i.e. the magneto-structural partly coupled state, showing an intersection point between two transitions, as shown in the low field *M*(*T*) curves (Fig. 1, site ③). Furthermore, the separation of the heating and cooling *M*(*T*) curves during SOT (labeled as red arrow in Fig. 1) also implies the occurrence of the magneto-structural partial coupling. That is to say, during cooling, a fraction of the paramagnetic austenite transformed to its ferromagnetic state while the rest may directly transformed to the ferromagnetic martensite (7 M structure with crystal lattice constants *a* = 4.26 Å, *b* = 5.54 Å, *c* = 42.26 Å, *β* = 93.1°, attained from Fig. 3b).

**Figure 5:**
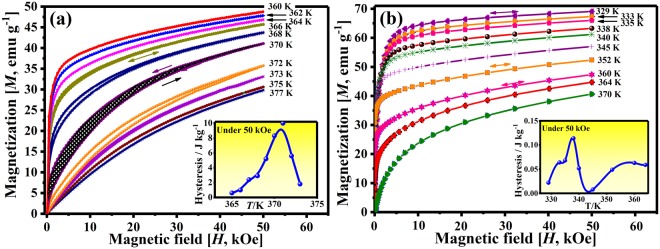
Magnetization isotherms of (**a**) W1 and (**b**) W2 showing the hysteresis character of the Ni-Mn-Ga microwires. The arrows indicate the direction change of the applied field, and the shade area represents the hysteresis loss for one particular isotherm cycle. The insets in (**a** and **b**) show the hysteresis loss at 50 kOe as a function of temperature for the displayed magnetization isotherms of W1 and W2, respectively.

The *ΔS*_*m*_(*T*) of W2 under different magnetic fields are shown in Fig. 4b. Maximum *ΔS*_*m*_ values of ~−5.3 J kg^−1^ K^−1^ related to FOT and ~−5.2 J kg^−1^ K^−1^ attributed to SOT are obtained at 50 kOe, respectively. Small positive peaks with values up to ~0.14 J kg^−1^ K^−1^ near FOT were also detected under magnetic fields lower than 3 kOe (marked as arrow in Fig. 4b). The critical field and positive peak values of W2 are relatively higher than those of W1 mainly due to the larger difference between *T*_*c*_ and *M*_*p*_ (*e*/*a* = ~7.66)^25^. Of note is that the transition region, shown as the “valley” between the two peaks, exhibits a minimum *ΔS*_*m*_ value of ~−4.0 J kg^−1^ K^−1^ under 50 kOe. This saddle-shaped peak gives rise to an extremely wide *WTI*, i.e. FWHM of the *ΔS*_*m*_(*T*) peak, of ~60 K (marked in Fig. 4b). On the other hand, according to the field-up and field-down isothermal magnetization curves (Fig. 5), negligible hysteresis losses are found in W1 (~4.5 J kg^−1^) and W2 (~0.08 J kg^−1^) by averaging the integral area under the working temperature interval (insets in Fig. 5). These vanishingly small hysteresis losses (Supplementary Information Table S3) are mainly attributed to the reduced resistance of the magnetic domain wall motion because of the high SSA of microwire and low internal stress and defect density after annealing^13^. Combined with the enhanced *WTI* and the reduced hysteresis, an increased *RC* value is thus expected in W2.

The *RC* value is a measure of transport of thermal energy between hot and cold reservoirs in one ideal refrigerate cycle, which can be calculated by integrating the *ΔS*_*m*_(*T*) curve over the FWHM range (*T*_1_ − *T*_2_) (Equation (2)).

2 $$RC={\int }_{{T}_{1}}^{{T}_{2}}{\rm{\Delta }}{S}_{m}{(T)}_{H}dT$$

After subtracting the average hysteresis losses, the net refrigeration capacity (*RC*_*net*_) values as a function of magnetic field change for the present W1 and W2 are obtained (Supplementary Information Fig. S7). The sharpened transition area leads to the low *RC*_*net*_ values of W1 (~25.1 and ~63.6 J kg^−1^ for 20 and 50 kOe, respectively). While in the partly coupled state, the *RC*_*net*_ values of W2 (~91.5 and ~240.0 J kg^−1^ for 20 and 50 kOe, respectively) show greater advantages than those of W1 and other Ni-Mn-based bulk or small-sized alloys (Supplementary Information Fig. S7).

**Figure 6:**
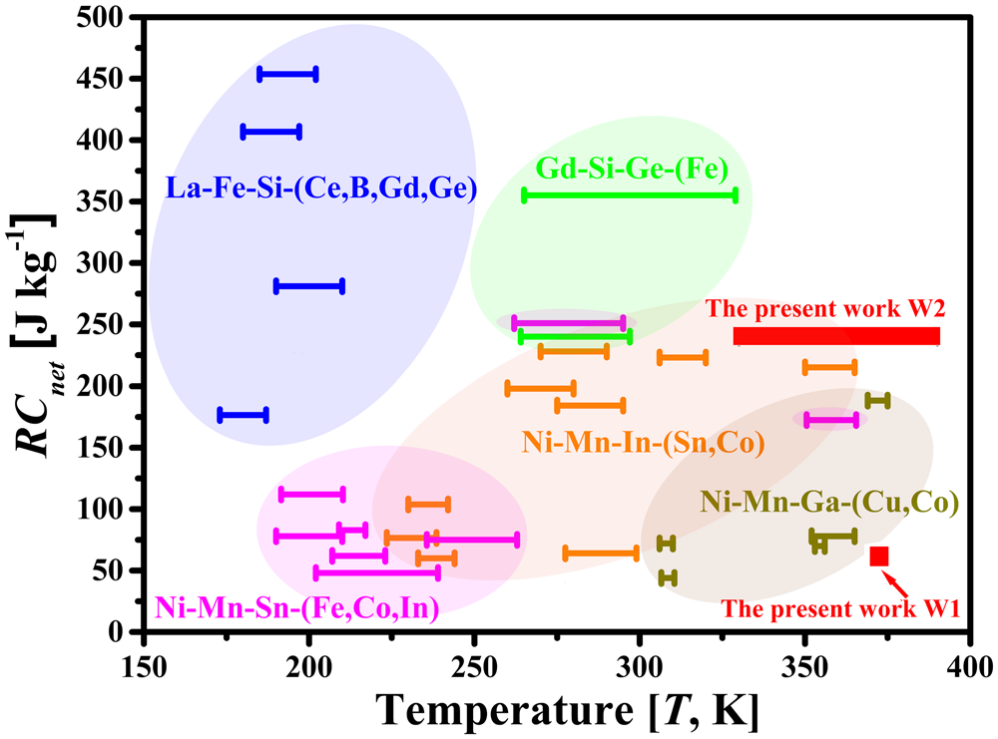
Schematic illustration of the net refrigeration capacity (*RC*_*net*_) as a function of temperature under a magnetic field of 50 kOe for the most studied magnetocaloric materials (Ni-Mn-Sn-based^7,27–32^, Ni-Mn-In-based^5,6,33–35^, Ni-Mn-Ga-based^2,3,11,36,37^, Gd-Si-Ge-based^38^, and La-Fe-Si-based^39,40^ alloys) and the present Ni-Mn-Ga microwires. The width of line segment indicates the working temperature interval of each alloy. (The ellipses only act as guide for the eye).

For comparison, the *RC*_*net*_ values as a function of temperature under a field of 50 kOe for the most studied MCE materials (Ni-Mn-Sn-based^7,27–32^, Ni-Mn-In-based^5,6,33–35^, Ni-Mn-Ga-based^2,3,11,36,37^, Gd-Si-Ge-based^38^, and La-Fe-Si-based^39,40^ alloys) are schematically illustrated in Fig. 6. Clearly, the present W2 shows the largest *WTI* values and considerable *RC*_*net*_ among the Ni-Mn-based MCE materials, and is comparable to that of the most promising rare-earth Ga-Si-Ge and La-Fe-Si based MCE materials. Furthermore, when compared to rare-earth compounds, Ni-Mn-Ga microwires are also rare-earth free and cost effective, which are beneficial to the practical applications.

In summary, the present study highlights that, other than many complex approaches, a feasible solution of preparing conventional Ni-Mn-Ga one-dimensional microwires with tunable transition temperatures can enhance the MCE and improve the practicability of the refrigeration materials. On the one hand, the one-dimensional micron-sized wires exhibit relatively good mechanical stability than bulk alloys, which is important in practical applications. On the other hand, regarding the MCE, tunable transition temperatures give rise to the possibility to obtain the designed transition states, including magneto-structural fully coupled state which results in large *ΔS*_*m*_, and partly coupled state which leads to extended *WTI* and high *RC*. Furthermore, the MT temperature range of the microwires can be tuned by creating a gradient composition distribution state of Mn through high temperature vacuum annealing. This criterion is applicable for other small-sized materials, such as ribbons and powders, whose FOT and SOT may be tuned by volatilization of one or more elements during high temperature annealing. Moreover, considering that high heat exchange efficiency is necessary for magnetic refrigeration, microwires show advantages over bulk alloys because of the higher SSA of the former. The microwires may be directly used as micron-sized devices or act as building blocks for minor- and macro-devices. Therefore, the combination of the tunable MCE property, large *WTI*, low thermal/magnetic hysteresis loss, high heat exchange efficiency, reasonably good mechanical stability and low cost renders Ni-Mn-Ga microwires valuable advantages, making them highly promising for the magnetic refrigeration.

## Materials and Methods

### Microwire preparation and heat treatment

The Ni-Mn-Ga master alloy ingots with nominal composition of Ni_50.5_Mn_29.5_Ga_20_ (C1) and Ni_50.6_Mn_28_Ga_21.4_ (C2) were prepared by induction melting pure Ni (99.99%), Mn (99.98%) and Ga (99.99%) under argon atmosphere and vacuum casting into a copper mold with a diameter of 9.0 mm. The fabrication process of Ni-Mn-Ga microwires by melt-extraction technique has been reported in ref.^13^ of our previous work. Three different microwires were prepared after chemical ordering and stress relief annealing (denoted as W1, W2 and W3, where W1 comes from nominal composition C1, W2 and W3 both from C2, respectively): (1) For W1 and W3, the as-extracted microwires were sealed in a quartz ampule, back-filled with 0.5 atm. pure Ar atmosphere, stepwise heat-treated at 998 K for 2 h, 973 K for 10 h and 773 K for 20 h and furnace cooled. To avoid oxidation and Mn vaporization during the heat-treatment, pure Ti foils and Mn particles were sealed in the tube along with the microwires; (2) For W2, vacuum heat treatment (same steps as in 1) but without Mn particle addition was applied in order to create a gradient Mn distribution state during annealing heat treatment. As for the stepwise heat treatment process, “998 K for 2 h” is a chemical ordering annealing process for eliminating the vacancies and atomic disorder formed during melt-extraction thus creating a chemical ordered structure, and “973 K for 10 h” is for consolidating the chemical ordered state. “773 K for 20 h” is a stress relief annealing process for releasing the internal stresses formed during melt-extraction.

### Composition and martensite transformation tests

The compositions were determined by a Zeiss-SUPRA SEM equipped with an Oxford EDS using 20 kV voltage, 97 µA emission current, 10 mm work distance and 50 µA probe current and >60 s data acquisition time duration. The composition measurement precision of the EDS was calibrated with chemical analysis results (ICP-OES) to be less than 0.5%. Ni_50.4±0.2_Mn_29.4±0.1_Ga_20.2±0.2_ (W1) and Ni_50.6±0.2_Mn_28.1±0.6_Ga_21.3±0.5_ (W2) microwires were designed to study the MCE. The characteristic MT temperatures were measured by a TA Q2000 differential scanning calorimeter (DSC) with cooling and heating rates of 5 K min^−1^. The crystal structures of the microwires were determined in a Rigaku D/max-γA X-ray diffractometer (XRD) with Cu Kα radiation (λ = 1.54 Å) at room temperature. A bunch of parallel microwires were placed on a monocrystalline silicon wafer for XRD measurement in order to increase the diffraction intensity and facilitate the operation.

### Magnetic property and magnetocaloric effects (MCE) evaluation

The magnetization measurements were carried out using a vibrating sample magnetometer (VSM) in a commercial Magnetic Property Measurement System (MPMS) of Quantum Design, where the magnetic field was applied along the longitudinal direction of the wire samples in order to minimize the effect of the internal demagnetization field. The isofield magnetization curves *M*(*T*) were measured with cooling and heating rates of 3 K min^−1^ under constant fields. The isothermal magnetization (*M-H*) curves for W1 were measured at different test temperatures (*T*_*test*_) from 377 to 354 K under an external magnetic field up to 5.0 T. In order to rule out the temperature and field history effects and thus avoid the spurious spike for the first order magnetic transition, the so-called loop process^41^ was performed before each *M-H* test. The detailed loop process was demonstrated as follows: (1) The sample was initially zero-field-heated up to 395 K to ensure a full paramagnetic austenite state prior to recording each *M-H* cycle at a constant temperature, (2) Zero-field-cooled to (*T*_*test*_-10) K at 10 K/min, then cooled to *T*_*test*_ at 1 K/min and finally maintained at the *T*_*test*_ temperature for 5 min before starting the *M-H* cycle. The *M-H* curves for W2 were measured on heating procedure from the temperature below *M*_*f*_ to temperature above *T*_*c*_^*A*^ (from 305 to 395 K) under an external magnetic field up to 50 kOe.

### Mechanical property tests

Ni_49.9±0.3_Mn_28.5±0.5_Ga_21.6±0.5_ microwires (W3) with MT temperatures near room temperature (RT) were used to facilitate the measurements of the mechanical stability. Stress-assisted thermal cycling and superelastic cycling were performed on a Q800 DMA with tension mode. To determine the precise MT temperatures of each microwire, temperature dependences of the low-frequency elastic modulus and internal friction, *Tanδ*, were performed in the dynamic regime. The same wire was then subjected to thermal cycling under different stresses using two methods: (1) measurement on the DMA equipment with cooling and heating rate of 5 K min^−1^: the microwire was placed on the tension clamps, heated up to 353 K and held for 12 min, then a stress was applied and subsequently cooled to 243 K, kept for 12 min and then heated to 353 K; this procedure was repeated for several times or changed to another external stress cycle, (2) manually heated the wire with hot-air blower and cooled with liquid nitrogen while kept the external stress intact at 65 and 100 MPa. The thermal cycles were repeated twice for each stress using method (1), and more than 100 times of cycles were applied using method (2). Superelastic loops were performed at 300 K: the microwire was heated up to 353 K and kept at this temperature for 12 min, then cooled to 300 K (slightly higher than *M*_*s*_) and subjected to a tensile loading-unloading cycle with a rate of 15 and 30 MPa min^−1^, respectively. After each cycle at 300 K, the microwire was heated to 353 K and kept for 12 min. Then the microwire was cooled to 300 K for a next cycle. The process was repeated for 103 times.

## Electronic supplementary material

Supplementary material-SREP-18-27909B
