# Supplementary material for: Tunable Magnetocaloric Effect in Ni-Mn-Ga Microwires
Source: Sci Rep. 2018 Nov 8;8:16574. doi: 10.1038/s41598-018-35028-9 (PMC6224590; doi:10.1038/s41598-018-35028-9)
Supplement: Supplementary file 1 — Supplementary material-SREP-18-27909B [file 41598_2018_35028_MOESM1_ESM.docx]

**Supplementary Information**

**Tunable Magnetocaloric Effect in Ni-Mn-Ga Microwires**

*Mingfang Qian, Xuexi Zhang^*^, Longsha Wei, Peter Martin, Jianfei Sun, Lin Geng, Thomas Scott, Hua-Xin Peng*

**
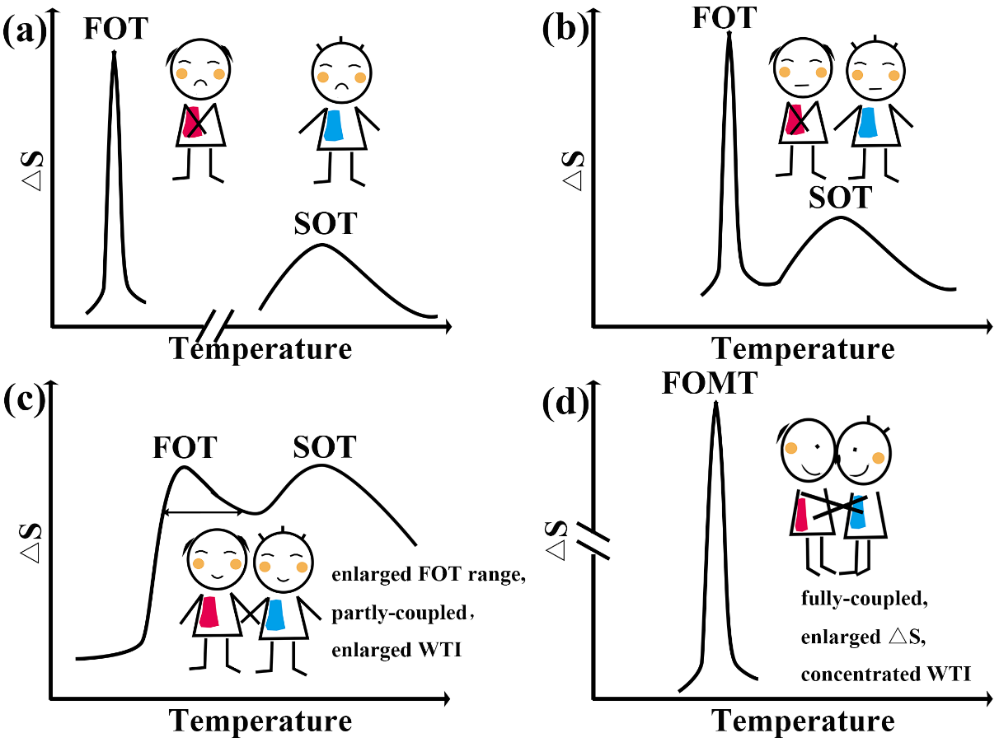
** **
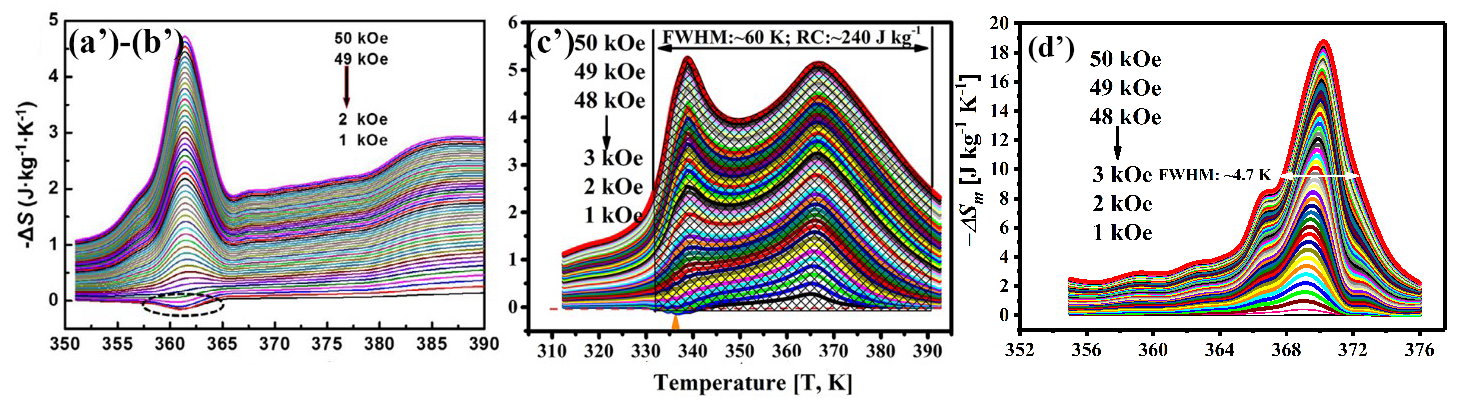
**

**Figure S1.** (a-d) Schematic illustrations and (a’-d’) corresponding temperature dependence of the calculated *ΔS_m_(T)* under different magnetic fields showing the effect of the temperature difference between the first-order martensite transformation (FOT) and second-order magnetic transition (SOT) as well as the FOT range on the magnetocaloric effect (MCE). (a) far apart, (b) getting closer, (c) partly coupled and (d) fully coupled. The widened FOT temperature range resulting in a partly coupled state decreases the magnitude of the *ΔS_m_* value compared to (d) while enlarges the *WTI*, as demonstrated in (c). The fully-coupled state in (d) significantly enhances the *ΔS_m_* while possessing extremely concentrated *WTI*. (**Figure (a’)-(b’)** is our previous work which has been published in ref S1, **Figure (c’)** and **(d’)** are reproduced from **Figure 4** in the manuscript just for convenience)


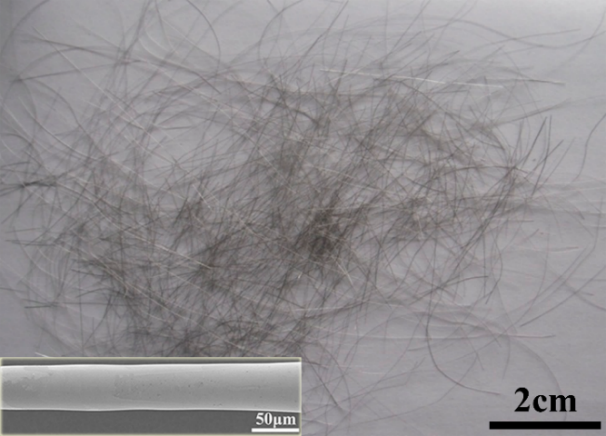

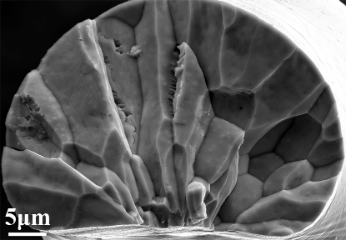


**(d)**

**(c)**


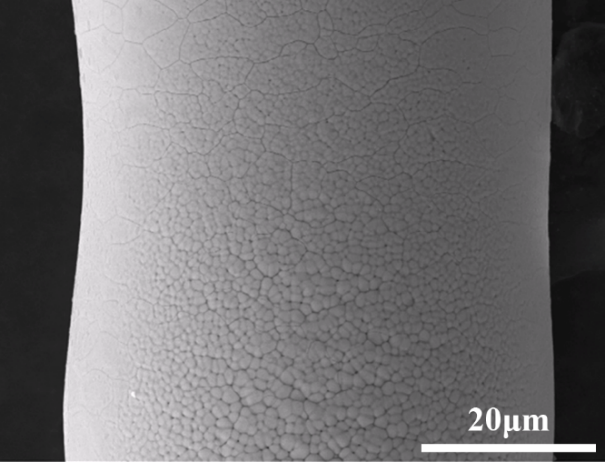

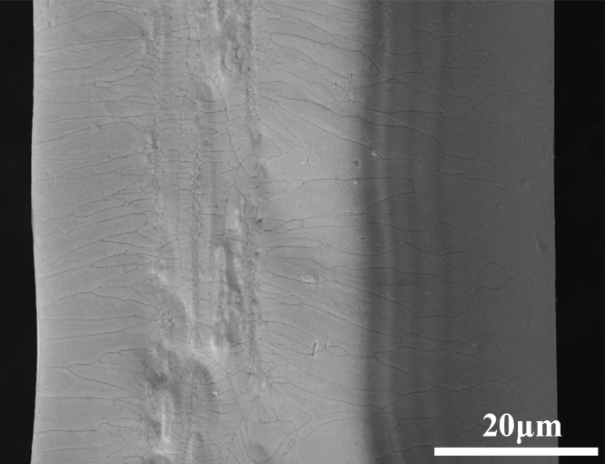


**(f)**

**(e)**


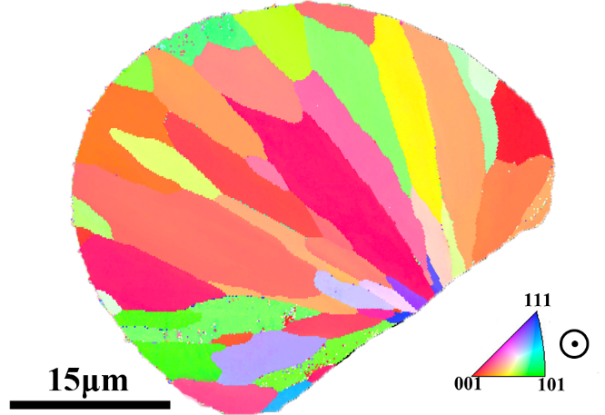

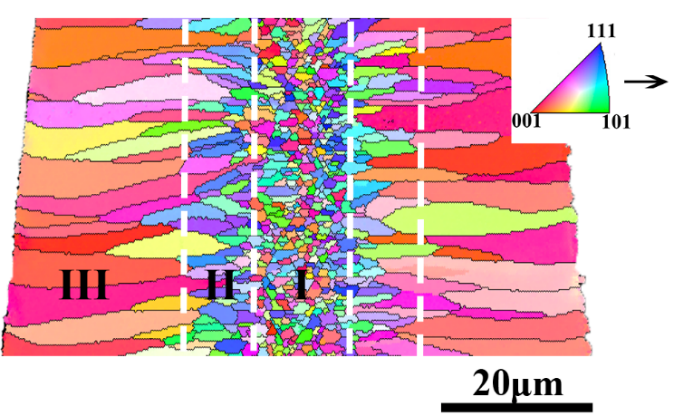


**Figure S2.** Typical morphologies of melt-extracted Ni-Mn-Ga microwires. (a) Macroscopic morphology, SEM image of the (b) cross-section, (c) free surface and (d) flattened surface of the microwire. EBSD (e) cross-section orientation maps (IPF normal direction coloring scheme) and (f) longitudinal section orientation maps (IPF coloring scheme relative to the in-plane direction perpendicular to the wire axis, near flattened surface) of the microwires. The orientation of each grain is according to the color of the EBSD maps.


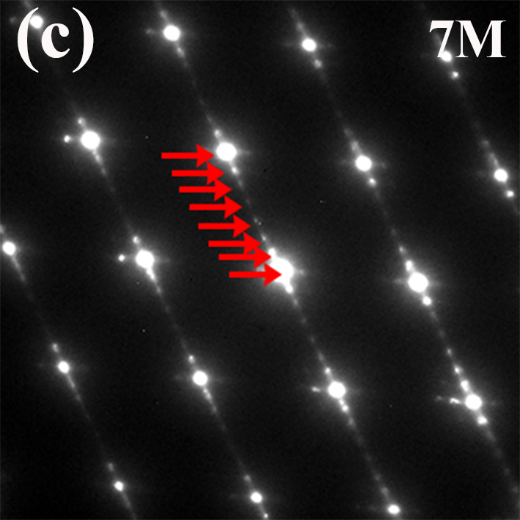


**Figure S3.** Typical electron diffraction patterns of W1 showing 6 extra spots between the main diffraction spots, confirmed the 7M structure in the microwire.

**Table S1.** Transformation temperatures shifting of W1 and W2 (determined by VSM)

| Microiwires | *A_p_(0.2kOe)* | *A_p_(50kOe)* | *M_p_(0.2kOe)* | *M_p_(50kOe)* | *ΔT_0h_* | *ΔT_0c_* |
| --- | --- | --- | --- | --- | --- | --- |
|  | K | | | | | |
| W1 | 371.3 | 375.2 | 368.1 | 370.9 | 3.9 | 2.8 |
| W2 | 337.1 | 340.1 | 334.9 | 336.9 | 3.0 | 2.0 |

According to **Figure 1** in the manuscript, the changes of the peak temperatures (temperature with respect to the extreme values of the first derivative of *M (T)* curves) are summarized in **Table S1**, where *ΔT_0h_* (*ΔT_0c_*) is the temperature difference between *A_p_* (*M_p_*) at 0.2 and 50 kOe. The data demonstrate that the sensitivity of the MT temperature to the applied field *dA_p_/dH* are 0.08 and 0.06 K kOe^-1^ for W1 and W2, respectively.


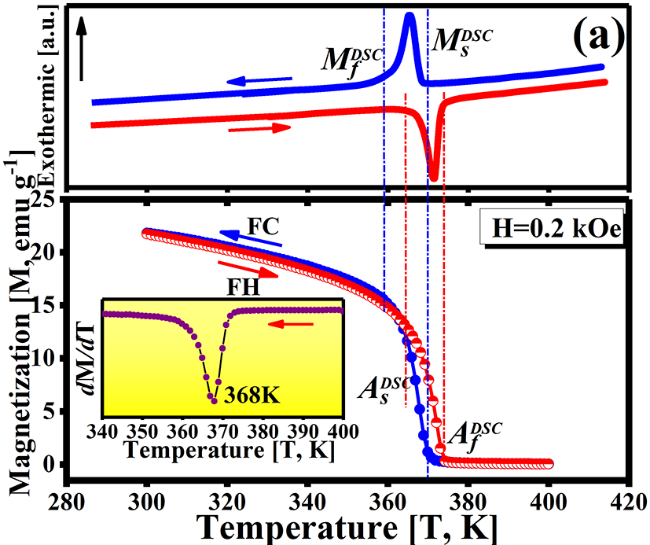

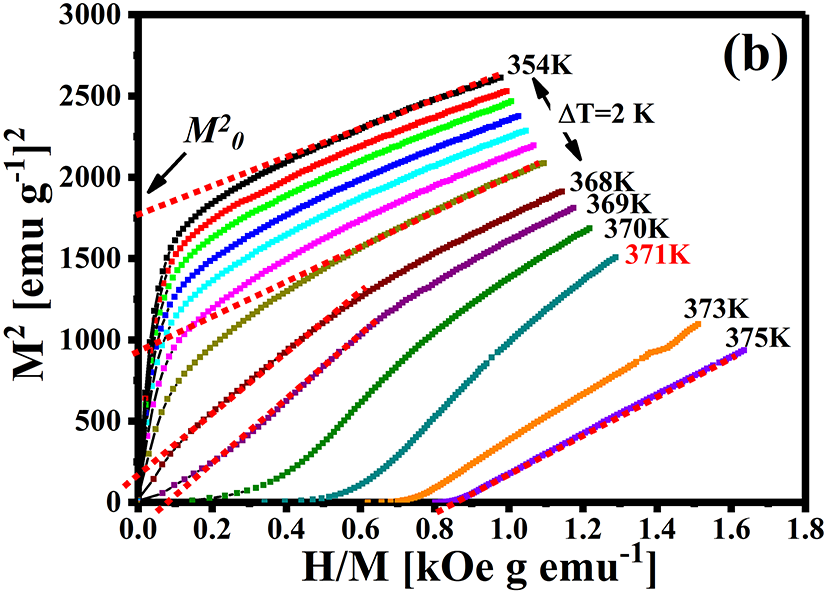

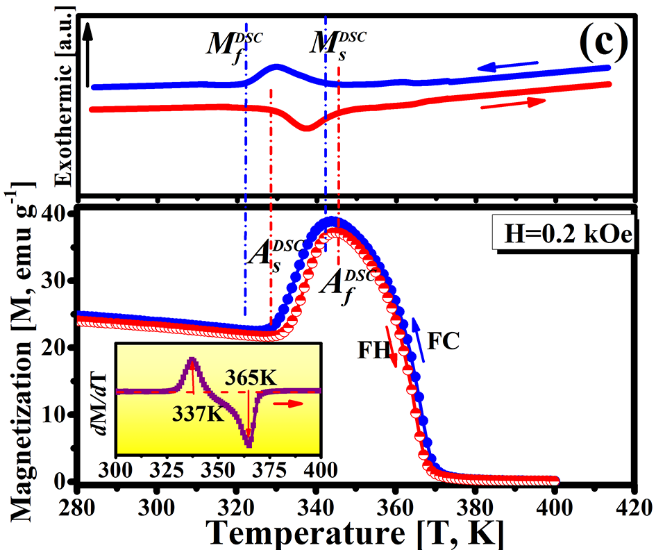


**Figure S4.** Heating and cooling DSC curves (upper) and the isofield magnetization *M(T)* curves of (a) W1 and (c) W2 at 0.2 kOe. The insets in (a) and (c) show the first derivative of the *M(T)* curves during heating. The vertical dashed lines correspond to the start and finish phase transformation temperatures (determined from the DSC scans) with the features of the low-field *M(T)* curves. (b) *M^2^ vs H/M* plots for W1 derived from the isothermal magnetization data for temperatures between 354 and 375 K. A linear extrapolation of curves is used to estimate the Curie temperature of the austenitic phase (*T_c_^A^*). The fact that $\text{M}_{\text{0}}^{\text{2}}$ is positive at 368 K but negative at 369 K indicates the onset of the ferromagnetic transition of the austenitic phase between them. A *ΔT_F-SOT_*, i.e. the temperature difference between the *M-T* curve first derivative extreme values of FOT (337K) and SOT (365K), ~28 K in W2 is attained.

**Table S2.** Martensitic transformation temperatures as well as enthalpy change values (determined by DSC) and Curie temperatures (determined by VSM) of W1 and W2.

| Microwires | *A_s_* | *A_p_* | *A_f_* | *M_s_* | *M_p_* | *M_f_* | *T_c_^A^* | *A_f_-A_s_* | **\|***Q_h_***\|** | **\|***Q_c_***\|** |
| --- | --- | --- | --- | --- | --- | --- | --- | --- | --- | --- |
|  | K | | | | | | | | J g^-1^ | |
| W1 | 366.6 | 371.4 | 373.7 | 368.6 | 365.4 | 361.1 | 368 | 7.1 | 6.8 | 7.1 |
| W2 | 329.8 | 336.6 | 345.0 | 342.4 | 330.2 | 321.8 | 365 | 15.2 | 4.5 | 4.6 |

The martensite and magnetic transition temperatures of W1 and W2 determined from **Figure S5a and c** are summarized in **Table S2**.

In Ni-Mn-Ga alloys, both Curie and MT temperature can be tuned over a wide range through the adjustment of the electron concentration *e/a* associated with a particular alloy composition^[S2]^. For *e/a* < 7.7, martensitic transformation occurs at the temperature far below the *T_c_^A^* and the reverse tendency occurs for large one *e/a* > 7.7. The intermediate range *e/a* = 7.7, where the two transitions overlap, is therefore of the great interests to MCE applications. After tuning the composition of the Ni-Mn-Ga alloys during induction melting, melt-extraction and subsequent chemical ordering annealing, W1 (*e/a* = 7.709) with coupled state and W2 (*e/a* = 7.709) with partly-coupled state were fabricated and investigated.

The transformation entropy change *ΔS_tr_* can be estimated as *ΔS_tr_^endo^* = **|***Q_h_***|**/*A_p_* and *ΔS_tr_^exo^*= **|***Q_c_***|**/*M_p_* during heating and cooling, respectively, where *Q* is the enthalpy change of the transformation. According to the DSC results, *ΔS_tr_^endo^* and *ΔS_tr_^exo^* can be calculated as ~18.3 and ~19.4 J kg^-1^ K^-1^ for W1 and ~13.4 and ~13.9 J kg^-1^ K^-1^ for W2, respectively.


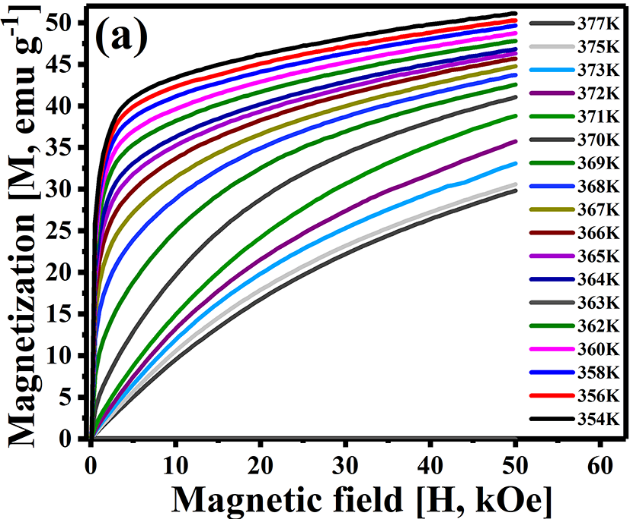

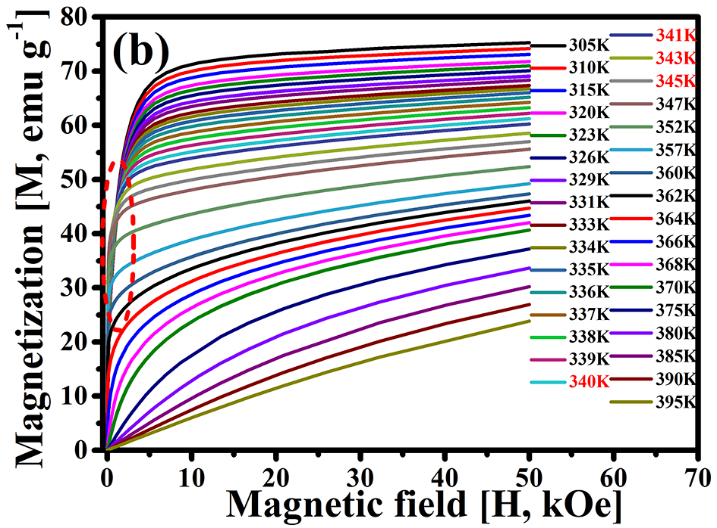


**Figure S5.** Isothermal magnetization *M(H)* curves of (a) W1 in the temperature range from 377 to 354 K and (b) W2 in the temperature range from 305 to 395 K.

As shown in the **Figure 4b** in the manuscript, tiny positive value of *ΔS^max^* appears as marked in yellow arrow. The value is ~0.14 J kg^-1^ K^-1^ under 1 kOe, then decreases and turns from positive to negative when the field is higher than 3 kOe. The reason for these phenomenon can be explained by the *M(H)* curves at extremely low field. As shown in **Figure S5b** by a dashed ellipse, the intersections between the *M(H)* curves at low field explained the positive to negative transition behavior of the entropy change in Ni-Mn-Ga microwires. This can be explained as follows: the magnetic moment in each magnetic domain tends to be in its stable state where all the moments have their preferred orientation in different domains, and the states are varied with temperatures. The magneto-crystalline anisotropy energy increases with decreasing temperature, causing the microwires harder to be magnetized at lower temperature, thus, leading to the intersections in **Figure S5b**.

More precisely, the positive part of MCE is originated from the magnetostructural coupling on the mesoscopic scale between the magnetic moments and martensite variants, which are also responsible for the magnetic shape memory effect^[S3]^. This part of the MCE occurs when the magnetic field is less than the saturate field of the martensite, and is proportional to the value of the saturate magnetization of the martensite. It is demonstrated that both values reach their maximum when close to the stoichiometric Ni_2_MnGa composition (*e/a*=7.5), and decrease with increasing *e/a*, that is, as *T_c_*-*T_m_* goes to zero. Therefore, the positive part is extremely small in W1 (0.02 J kg^-1^ K^-1^) with an e/a value of ~7.7, while relatively higher in W2 (0.14 J kg^-1^ K^-1^) with an e/a value of ~7.66.


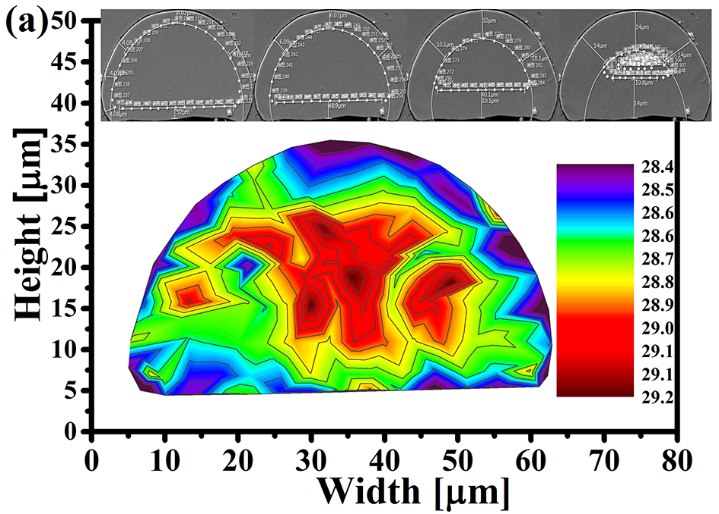

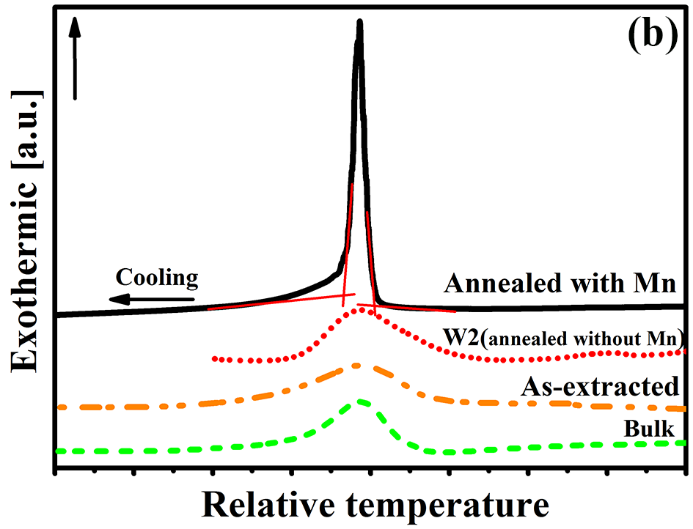


**Figure S6.** (a) Compositional distribution data (Mn element) in the cross-section of W2 after annealing according to the Experimental details. (b) Centered cooling DSC curves with respect to the ingot, the as-extracted and the annealed microwires with and without Mn particle addition. Curves are centered to facilitate the MT temperature range comparison.

In order to increase the magnetization, microwires are often subjected to a chemical ordering annealing. However, the chemical ordering annealing process always sharpens the transformation process and increases the magnetization, thus, may increase the MCE peak value but reduce the temperature span. In general, in order to prevent Mn vaporization of the microwire, Mn particles was set to seal with the microwires. Here for W2, the chemical ordering annealing without Mn particle addition was applied with the aim to increase the MT transformation range. Due to the high SSA of the microwires and high vaporization pressure of the Mn, Mn content variation from the wire surface to the inner part was created in the microwires, leading to a wide transformation range due to the strong dependence of the transformation temperature on the composition. Accompanying with the tuned temperature differences between FOT and SOT, a FOT and SOT partly coupled state can be obtained, as schematically shown in **Figure S1c.**

**Table S3.** Comparison of the thermal and magnetic hysteresis loss in Ni-Mn-based Heusler alloys and some rare-earth compounds used for magnetic refrigeration materials^[S1,S4-S18]^.

| Materials | Thermal hysteresis (K) | Magnetic hysteresis at 50 kOe  (J kg^-1^) | References |
| --- | --- | --- | --- |
| W1 | ~2.8 | ~4.5 | Present |
| W2 | ~1.3 | ~0.08 | Present |
| Ni_49.4_Mn_26.1_Ga_20.8_Cu_3.7_(W) | ~4.0 | - | ^[S4][1]^ |
| Ni_48_Mn_26_Ga_19.5_Fe_6.5_(W) | ~4.0 | - | ^[S1][2]^ |
| Ni_44.9_Fe_4.3_Mn_38.3_Sn_12.5_(W) | ~14.5 | ~32.9 | ^[S5][3]^ |
| Ni_45.8_Fe_4.2_Mn_38.0_Sn_12.0_(B) | ~17.5 | ~115 | ^[S6][4]^ |
| Ni_45.0_Mn_38.6_In_11.3_Co_5.1_(B) | ~24 | - | ^[S7][5]^ |
| Ni_47.7_Mn_37.1_In_15.2_(B) | ~10 | - | ^[S8]^ |
| Ni_49.0_Mn_38.5_Sb_12.5_(B) | ~18.5 | ~11.4 | ^[S9]^ |
| Ni_49.0_Mn_38.4_Sb_11.7_Si_0.9_(B) | ~13.0 | ~2.1 | ^[S9]^ |
| Ni_52_Mn_26_Ga_22_(R) | ~10 | ~11.3 | ^[S10]^ |
| Ni_55.8_Mn_18.1_Ga_26.1_(B) | ~10 | - | ^[S11]^ |
| Ni-Co-Mn-Sn (R) | ~10-16 | ~13.71-18.76 | ^[S12]^ |
| Ni_50_Mn_34_In_16_(B) | - | ~77.01 | ^[S13]^ |
| Ni_50.3_Mn_35.5_Sn_14.4_(B) | ~21 | ~8.33 | ^[S14]^ |
| Mn_50_Ni_40_In_10_(B) | ~18.6 | ~53.06 | ^[S16]^ |
| Gd_5_Si_1.8_Ge_1.8_Sn_0.4_(B) | ~9.2 | ~62 | ^[S16]^ |
| Gd_5_Si_1.8_Ge_1.8_Sn_0.4_(R) | ~3.8-4.2 | ~5.7-7.5 | ^[S16]^ |
| LaFe_11.6_Si_1.4_(F) | < 0.5 | - | ^[S17]^ |
| LaFe_11.57_Si_1.43_(F) | ~3 | - | ^[S18]^ |

**microwires (W), Foam (F), ribbons (R), bulk (B)*

**
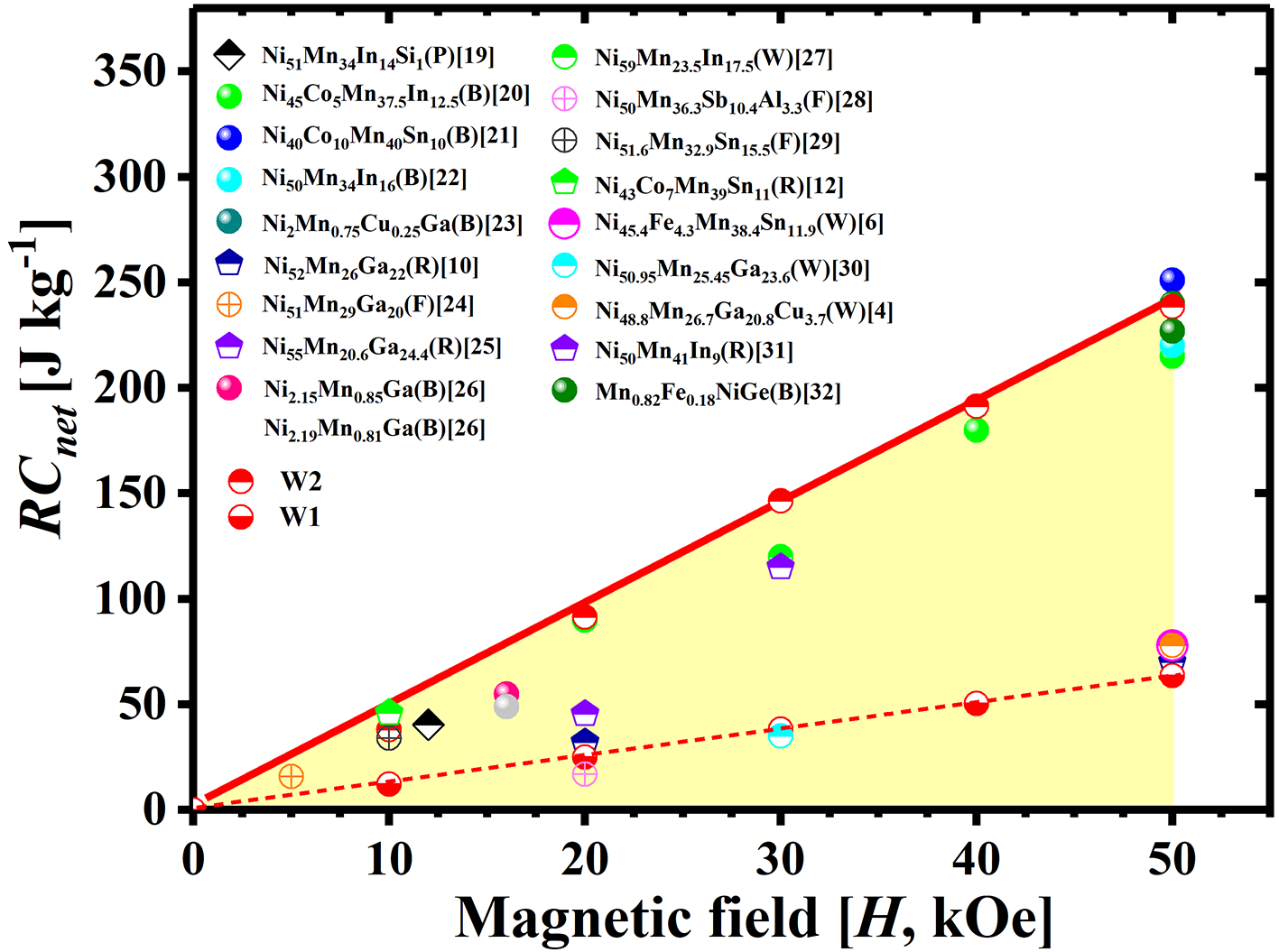
**

**Figure S7.** (Color online) Magnetic field dependence of the *RC_net_* value. Results of the present W1 and W2 microwires are compared to some Ni-Mn-based bulk and small-sized alloys ^[S4,S6,S10,S12,S19-32]^. The red lines (solid and dash) act as guide for the eye. (microwires (W), thin film (F), ribbons (R), bulk (B) and particle (P))

**References**

[S1] Y. Liu, X. Zhang, D. Xing, H. Shen, D. Chen, J. Liu, J. Sun, *J. Alloy. Compd.* **2014**, *616*, 184.

[S2] V. A. Chernenko, E. Cesari, V. V. Kokorin, I. N. Vitenko, *Scripta Mater.* **1995**, *33*, 1239.

[S3] J. Marcos, L. Manosa, A. Planes, F. Casanova, X. Batlle, A. Labarta, *Phys. Rev. B* **2003**, *68*.

[S4] X. X. Zhang, M. F. Qian, Z. Zhang, L. S. Wei, L. Geng, J. F. Sun, *Appl. Phys. Lett.* **2016**, *108*, 052401.

[S5] H. H. Zhang, M. F. Qian, X. X. Zhang, S. D. Jiang, L. S. Wei, D. W. Xing, J. F. Sun, L. Geng, *Mater. Design* **2017**, *114*, 1*.*

[S6] H. H. Zhang, M. F. Qian, X. X. Zhang, L. S. Wei, F. Y. Cao, D. W. Xing, X. P. Cui, J. F. Sun, L. Geng, *J. Alloy. Compd.* **2016**, *689*, 481.

[S7] X. X. Zhang, M. F. Qian, S. P. Miao, R. Z. Su, Y. F. Liu, L. Geng, J. F. Sun, *J. Alloy. Compd.* **2016**, *656*, 154.

[S8] X. X. Zhang, M. F. Qian, R. Z. Su, L. Geng, *Mater. Lett.* **2016**, *163*, 274*.*

[S9] R. C. Zhang, M. F. Qian, X. X. Zhang, F. X. Qin, L. S. Wei, D. W. Xing, X. P. Cui, J. F. Sun, L. Geng, H. X. Peng, *J. Magn. Magn. Mater.* **2017**, *428*, 464.

[S10] Z. Li, Y. Zhang, C. F. Sánchez-Valdés, J. L. Sánchez Llamazares, C. Esling, X. Zhao, L. Zuo, *Appl. Phys. Lett*. **2014**, *104*, 44101.

[S11] Z. Li, K. Xu, Y. Zhang, C. Tao, D. Zheng, C. Jing, *Sci. Rep.* **2015**, *5*, 15143*.*

[S12] S. C. Ma, C. W. Shih, J. Liu, J. H. Yuan, S. Y. Lee, Y. I. Lee, H. W. Chang, W. C. Chang, *Acta. Mater.* **2015**, *90*, 292*.*

[S13] V. K. Sharma, M. K. Chattopadhyay, S. B. Roy, *J. Phys. D: Appl. Phys.* **2007**, *40*, 1869.

[S14] B. Hernando, J. L. Sánchez Llamazares, J. D. Santos, V. M. Prida, D. Baldomir, D. Serantes, R. Varga, J. González, *Appl. Phys. Lett*. **2008**, *92*, 132507.

[S15] J. Ren, H. Li, S. Feng, Q. Zhai, J. Fu, Z. Luo, H. Zheng, *Intermetallics* **2015**, *65*, 10.

[S16] T. Zhang, Y. Chen, Y. Tang, *J. Phys. D: Appl. Phys.* **2007**, *40*, 5778*.*

[S17] J. Lyubina, R. Schäfer, N. Martin, L. Schultz, O. Gutfleisch, *Adv. Mater.* **2010**, *22*, 3735.

[S18] A. Yan, K. H. Müller, O. Gutfleisch, *J. Appl. Phys.* **2005**, *97*, 36102.

[S19] R. Das, A. Perumal, A. Srinivasan, *J. Alloys Compd.* **2013**, *572*, 192.

[S20] D. Bourgault, J. Tillier, P. Courtois, D. Maillard, X. Chaud, *Appl. Phys. Lett*. **2010**, *96*, 132501.

[S21] L. Huang, D. Y. Cong, H. L. Suo, Y. D. Wang, *Appl. Phys. Lett.* **2014**, *104*, 132407.

[S22] V. K. Sharma, M. K. Chattopadhyay, R. Kumar, T. Ganguli, P. Tiwari, S. B. Roy, *J. Phys. Condens. Mat.* **2007**, *19*, 496207.

[S23] S. Stadler, M. Khan, J. Mitchell, N. Ali, A. M. Gomes, I. Dubenko, A. Y. Takeuchi, A. P. Guimarães, *Appl. Phys. Lett.* **2006**, *88*, 192511.

[S24] Y. P. Zhang, R. A. Hughes, J. F. Britten, P. A. Dube, J. S. Preston, G. A. Botton, M. Niewczas, *J Appl. Phys.* **2011**, *110*, 13910.

[S25] N. V. R. Rao, R. Gopalan, V. Chandrasekaran, K. G. Suresh, *J. Alloy. Compd.* **2009**, *478*, 59.

[S26] L. Pareti, M. Solzi, F. Albertini, A. Paoluzi, *Eur. Phys. J. B* **2003**, *32*, 303.

[S27]V. Vega, L. Gonzalez, J. Garcia, W. O. Rosa, D. Serantes, V. M. Prida, G. Badini, R. Varga, J. J. Sunol, B. Hernando, *J. Appl. Phys.* **2012**, *112*, 033905.

[S28] R. Barman, D. Kaur, *Vacuum* **2015**, *120*, 22.

[S29] E. Yuzuak, I. Dincer, Y. Elerman, A. Auge, N. Teichert, A. Hutten, *Appl. Phys. Lett.* **2013**, *103*, 222403.

[S30] R. Varga, T. Ryba, Z. Vargova, K. Saksl, V. Zhukova, A. Zhukov, *Scripta Mater.* **2011**, *65*, 703.

[S31] H. Li, S. Feng, J. Ren, Q. Zhai, J. Fu, Z. Luo, H. Zheng, *J. Magn. Magn. Mater.* **2015**, *391*, 17.

[S32] R. Wu, F. Shen, F. Hu, J. Wang, L. Bao, L. Zhang, Y. Liu, Y. Zhao, F. Liang, W. Zuo, J. Sun,B. Shen, *Sci. Rep.* **2016**, *6*, 20993.
